# The Progress of Intestinal Epithelial Models from Cell Lines to Gut-On-Chip

**DOI:** 10.3390/ijms222413472

**Published:** 2021-12-15

**Authors:** Shafaque Rahman, Mohammed Ghiboub, Joanne M. Donkers, Evita van de Steeg, Eric A. F. van Tol, Theodorus B. M. Hakvoort, Wouter J. de Jonge

**Affiliations:** 1 Tytgat Institute for Liver and Intestinal Research, Amsterdam Gastroenterology, Endocrinology and Metabolism, Amsterdam University Medical Centers, Location AMC, University of Amsterdam, 1105 BK Amsterdam, The Netherlands; s.rahman@amsterdamumc.nl (S.R.); m.ghiboub@amsterdamumc.nl (M.G.); t.hakvoort@amsterdamumc.nl (T.B.M.H.); 2 Department of Pediatric Gastroenterology and Nutrition, Amsterdam University Medical Centers, Emma Children’s Hospital, 1105 AZ Amsterdam, The Netherlands; 3 The Netherlands Organization for Applied Scientific Research (TNO), 3704 HE Zeist, The Netherlands; joanne.donkers@tno.nl (J.M.D.); evita.vandesteeg@tno.nl (E.v.d.S.); ric.vantol@tno.nl (E.A.F.v.T.); 4 Department of Surgery, University of Bonn, 53113 Bonn, Germany

**Keywords:** intestine, in vitro, ex vivo, organoids, gut-on-chip, inflammation

## Abstract

Over the past years, several preclinical in vitro and ex vivo models have been developed that helped to understand some of the critical aspects of intestinal functions in health and disease such as inflammatory bowel disease (IBD). However, the translation to the human in vivo situation remains problematic. The main reason for this is that these approaches fail to fully reflect the multifactorial and complex in vivo environment (e.g., including microbiota, nutrition, and immune response) in the gut system. Although conventional models such as cell lines, Ussing chamber, and the everted sac are still used, increasingly more sophisticated intestinal models have been developed over the past years including organoids, InTESTine™ and microfluidic gut-on-chip. In this review, we gathered the most recent insights on the setup, advantages, limitations, and future perspectives of most frequently used in vitro and ex vivo models to study intestinal physiology and functions in health and disease.

## 1. Introduction

The intestine plays a crucial role in regulating systemic physiology through food digestion and uptake, drug transport, metabolism, excretion [1], and hormone secretion [2,3,4]. Furthermore, the gut wall is inhabited by large microbial communities, which help to maintain intestinal homeostasis by playing an essential role in the production of short chain fatty acids and vitamin K and as well as in drug metabolism, for instance [5]. A dysbiotic state of the gut microbiome has been associated with several diseases, including IBD, irritable bowel syndrome (IBS), and colorectal cancers [6,7,8]. To study intestinal processes in a laboratory setting, a wide range of in vitro and ex vivo models have been developed over the years, that aim to look into gut physiology, intestinal barrier function, drug absorption, and host–microbe/cell–cell co-culture in both healthy and diseased conditions. In this review, we summarize the conventional and more recently developed in vitro and ex vivo systems focusing on their applications, advantages, and limitations.

## 2. In Vitro and Ex Vivo Intestinal Models

### 2.1. In Vitro Models

#### 2.1.1. Epithelial Cell Lines

Developed in the 1970s, both Caco-2 and HT-29 cell lines are derived from human colorectal adenocarcinoma [9], while the T84 cell line is known as a transplantable human colon carcinoma line developed in the 1980s [10]. Caco-2 and HT-29 cell lines possess similar enterocyte properties upon differentiation, but they also have some differences [11,12,13]. While Caco-2 cells differentiate towards an absorptive phenotype monolayer without mucus granules [12,14], thereby exhibiting lower permeability compared to the human intestine [15], HT-29 cells display high heterogeneity in absorptive and goblet cells formation and are consequently able to produce mucus [13]. Especially two of the HT-29 subclones (HT-29-H and HT-29-MTX) have gained importance in drug absorption and permeability studies because of their mucus-producing capabilities [16,17]. In addition to permeability studies in monocultures of Caco-2 and HT-29 cells, many drug absorption and transport studies are performed in Caco-2-HT-29-MTX co-cultures, thereby benefitting from the presence of the mucus layer produced by the latter [18,19,20,21]. For instance, the co-culture model was used to assess the permeability of twelve drugs belonging to different Biopharmaceutical Classification System (BCS) characteristics where these drugs showed higher permeability values in the co-culture model as compared to Caco-2 monocultures alone [21]. Additionally, co-culturing Caco-2 with HT-29-MTX cells lead to an increased apical to basolateral permeability of P-glycoprotein mediating intestinal secretion [22]. T84 is another cell line used to study intestinal permeability [23,24,25] and electrolyte transport mechanisms [26,27,28]. Similar to Caco-2 cells, T84 cells can spontaneously differentiate into an absorptive monolayer of epithelial cells [29]. T84 cells can also differentiate into crypt-like cells upon induction with mesenchymal soluble factors or human transforming growth factors [30]. One of the examples of the use of this cell line with regard to electrolyte transport was to determine the role of store-operated cAMP (SOcAMPs) signaling in the calcium activated secretion of chloride [27]. The activation of SOcAMPs could contribute to the various xenobiotic agents’ actions leading to diarrhea [27].

**Figure 1:**
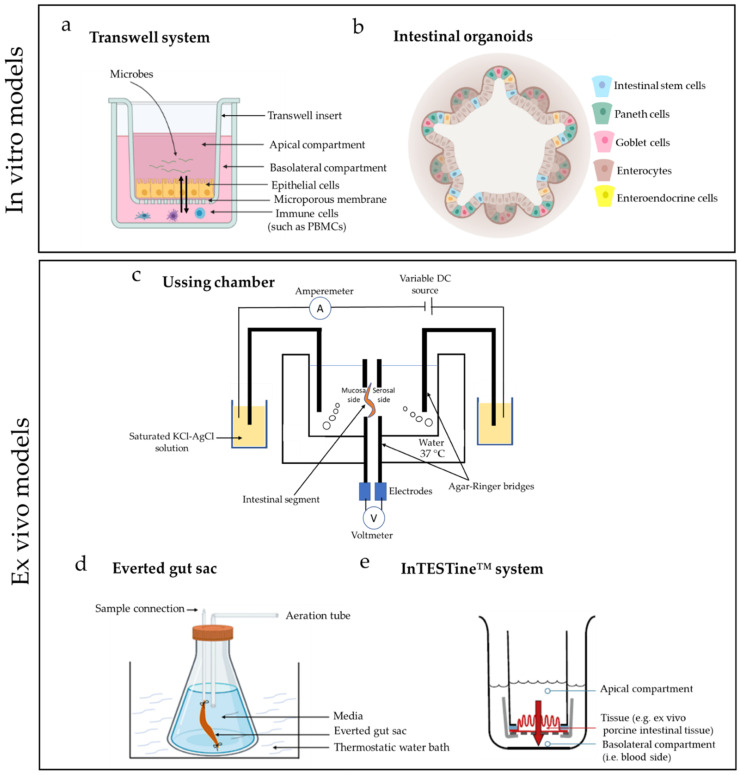
Illustration of in vitro and ex vivo models. Panel (**a**) schematic representation of a Transwell system, where epithelial cells, immune cells such as peripheral blood mononuclear cells (PBMCs), microbes, and other features can be co-cultured together. Panel (**b**) schematic representation of intestinal organoids highlighting the different cell types. Panel (**c**) shows the setup of Ussing chamber system where the temperature is maintained at 37 °C in the reservoir. Panel (**d**) is a representation of the everted gut sac system. The everted gut sac is transferred to the incubation flask containing oxygenated media. Panel (**e**) illustrates the designed system InTESTine^TM^ from TNO with apical and basolateral compartments [40]. Panel (**a**–**d**) illustrations were created with BioRender.com and permission of the authors were taken for using the illustration in panel (**e**).

Widely known for transport kinetic studies [31,32,33,34], the polarized orientation of Caco-2 cells is especially useful for drug and nutrient transport studies, which need separation of the apical and basolateral sides to predict oral availability in vivo. With this goal in mind, the cells are typically grown on Transwell inserts (Figure 1a). Transwell inserts are commercially available plastic inserts that can be placed in a multi-well tissue culture plate, thereby establishing a two-chamber system which is separated by the permeable membrane of the insert [35]. The use of these inserts allows to investigate cell migration between the two chambers and provides access for the exchange of soluble factors between them [36]. As an example, transport of dietary anti-inflammatory peptide (γ-Glutamyl Valine) was assessed across Caco-2 monolayers grown onto Transwell inserts [37]. The Transwell model can also be used to assess barrier functions of cells by trans-epithelial electrical resistance (TEER). For instance, changes in TEER measurements were seen when co-culturing Caco-2 monolayers with *Bifido*- and/or *E. coli* bacteria [38]. Adhesion of *E. coli* to the epithelial layer led to a maximum increase of TEER after 4–8 h, while adhesion of bifidobacteria (in absence of *E. coli*) led to a delayed TEER maximum [38]. Although mostly used as a 2D model, Caco-2 cells can also be grown in 3D [39]. Cultured in 3D, the cells showed a higher expression of serotonin transporter (SERT), a promising therapeutic target for intestinal inflammation and diarrhea [39].

Additionally, co-cultures of intestinal epithelial and immune cells are studied such as Caco-2 cells in co-culture with the human monocytic cell line (THP-1) [41] and the monoblastic cell line (U937) [42]. Caco-2 cells that were first primed with IFN-γ and then co-cultured with LPS+IFN-γ stimulated human monocytic cell line (THP-1 cells) had reduced barrier integrity and showed expression of pro-inflammatory cytokines (TNF-α, IL-1β) and chemokine IL-8 [41]. Studies addressing the (host–microbe) immune response also benefit from using Transwell inserts where (non)-pathogenic bacteria can be applied at the apical side while cytokine secretion can consequently be measured at the basolateral side [43,44].

Thus, the intestinal cell lines are also used to study the interaction of the host (cells) with the gut microbiome. Caco-2 and HT-29 cells express toll-like receptors (TLRs) sensing bacterial antigens which makes these cell lines interesting for conducting host–microbiome studies [45]. For example, crosslinking of *Salmonella enterica sv. Typhimurium* to differentiated Caco-2 cells led to the discovery of four host receptors bound to previously not known ligands for the bacteria [46]. Caco-2 cells were also used to elucidate the anti-inflammatory effects of *Lactobacillus rhamnosus GG* and *Lactobacillus casei* [47]. These bacteria suppressed the release of *Escherichia coli (E. coli)* induced chemokines expression [47]. Furthermore, both Caco-2 and HT-29 cell lines have been used to show the protective effects of probiotics against pathogens [48]. For example, Resta-Lenert et al. have shown that exposing these epithelial cell lines with live probiotic *Streptococcus thermophilus* and *Lactobacillus acidophilus* limits the invasion and adhesion of enteroinvasive *Escherichia coli* (EIEC) [48]. Additionally, in T84 cells, the protective effects of Lactoferrin on bacteria-driven barrier dysfunction was studied in which treatment with Lactoferrin restored tight junction dysfunction during *Yersinia enterocolitis* infection [49]. Despite the most widespread use of the human intestinal epithelial cell lines, there are also some intestinal cell models of animal origin to study processes such as neonatal gut development as well as understanding host–microbiome interactions [50,51].

To study the different functionality of intestinal cells or an inflammatory environment under diseased conditions, e.g., in IBD, co-culture of Caco-2 and HT-29-MTX cells have proven to successfully mimic the intestinal inflammatory status in IBD as seen through reduced Zona occludens 1 (Zo-1) and increased mucin 2 (MUC2) expression [52]. In terms of other disease, such as sigmoid colon adenocarcinoma, the HT-29 cell line is particularly suitable to study monocarboxylate transporter 1 (MCT1) transporter function due to the consistently higher presence of the protein in the cell line [53]. These examples suggest the possibility to select different cell lines to study different diseases in an in vitro set-up.

Despite the many advantages of these above-mentioned human cell lines, i.e., high throughput, cost-effectiveness, and ease of use [54], they exhibit several limitations. For instance, Caco-2 cells lack the mucus-producing layer, and it is difficult to control its differentiation [55]. Additionally, Caco-2 cells exhibit different transporter expression as compared to human intestinal tissue [56]. The HT-29 cells show impaired glucose metabolism with high glucose consumption and glycogen accumulation [13,57]. Furthermore, HT-29 cells mimic some small intestine characteristics, but they are not comparable to the colonic enterocytes as HT-29 cells express brush border enzymes, amongst other hydrolases [13,57]. Lastly, the T84 cell line also has similar disadvantages to Caco-2 and HT-29 cell line due to its cancerous origin and lack of epithelium specific function seen in vivo.

#### 2.1.2. Intestinal Organoids

The introduction of organoids in research has provided an advanced tool to study various intestinal functions while keeping the epithelial cells in a more physiological condition [58]. Organoids are 3D micro-tissues, with the lumen inside of the 3D structure, and developed from different tissue sources. These mainly involve pluripotent stem cells, either embryonic stem cells or induced pluripotent stem cells [59,60,61], or multipotent stem cells which are then embedded in an extracellular matrix (ECM)-rich hydrogel provided with a culture medium containing essential growth factors [58,59,60]. These intestinal organoids not only contain diverse cell types but also expresses pharmacokinetic genes and drug metabolizing enzymes (such as CYP3A4) similar to organoids derived from human induced pluripotent stem cells [62]. The few essential components necessary to generate organoids include: (a) a wingless-related integration site (Wnt) source and R-spondin enhancing the Wnt signaling pathway, (b) epidermal growth factor (EGF), (c) inhibitor of bone morphogenetic protein (BMP)/TGF-β signaling, and (d) ECM-rich hydrogel [59]. An example of the intestinal organoid model is illustrated in Figure 1b.

Due to its potential to generate specific cell types, organoids are extensively used to study intestinal epithelium in vitro [63,64]. Although mostly grown in 3D, organoids can also be seeded and grown as monolayers that allow easier access to the apical side and studies towards the intestinal barrier function [65]. An example includes barrier function evaluation of a co-culture of human intestinal organoid monolayers and human monocyte-derived macrophages [66]. Barrier function was enhanced in this co-culture model as reflected by increased TEER measurements [66]. A study on barrier integrity also used mouse small intestinal organoids to evaluate the impact of IFN-γ induced disruption of tight junction proteins (claudin-2, -7, and -15) over time [67]. In addition, mouse organoids have been used to study other biological processes such as cell death and apoptosis, for instance through a modified MTT (3-(4,5-dimethylthiazol-2-yl)-2,5-diphenyltetrazolium bromide) assay [68]. MTT was used to stain the viable crypt organoids which helped in distinguishing viable from dead intestinal organoids using bright-field microscopy [68]. Furthermore, organoid technology has been used to study intestinal embryonic development [69].

With regards to the application of organoids to study diseases, organoid transplantation is suggested for the regeneration of refractive ulcers and reduction of colitis-associated cancers in IBD patients [70]. Furthermore, transplantation studies in mice have proven informative to understand the role of organoids in tissue repair [71,72]. In a study on the dextran sulfate sodium induced (DSS) colitis model, mice have shown the engraftment of organoids onto the rectal ulcer surface when delivered through the intraluminal route [71]. The donor-derived cells formed crypts that were integrated into the recipient epithelial crypts where they remained present for over 6 months [71]. Similar observations were seen using organoids derived from adult small intestine in mice [73], and further confirmation was made through the reconstruction of damaged mucosa of immunodeficient mice by human intestinal organoids (HIO) [72]. Additionally, organoids derived from colorectal cancer patients exhibited distinct markers of tumor growth such as an upregulated revival stem cell population marker called Clusterin. This marker was significantly enriched following the 5-fluorouracil (5-FU) chemotherapy treatment, which was correlated to lower patient survival and increased colorectal cancer progression [74]. Primary intestinal organoids derived from cystic fibrosis patients showed strong reduction in swelling when stimulated with forskolin; this was in contrast to the effect seen in organoids derived from human healthy controls or wild-type mice. The forskolin-induced swelling was also found to be CFTR (cystic fibrosis transmembrane conductance regulator) dependent. Therefore, organoids offer drug development and personalized medicine advances for cystic fibrosis [75].

Intestinal organoids are also used to study host–microbe interactions. As compared to cell lines, organoids can be a great asset to study the effect of microbial metabolites/toxins as such due to the presence of multiple cell types closely resembling the in vivo intestinal environment [58]. As an example of a host–microbe study, organoids were successfully applied to demonstrate the mechanisms of secretory diarrhea caused by *Vibrio cholera* [69,76]. The mechanism of diarrhea by cholera is suggested to be due to the luminal release of the cholera toxin, as cholera toxin exposure to organoids inhibited NHE3 (an ion transport protein) transporter activity and stimulated fluid secretion [69,76]. Another example includes the study involving *Shigella* (an invasive pathogen) pathogenesis using human intestinal organoids [77,78]. The organoid model has also been used to study viral, bacterial, and protozoan parasite infections [79,80,81,82,83]. In light with the recent development of the severe acute respiratory syndrome coronaviruses (SARS-CoV and SARS-CoV-2), epithelial intestinal organoids are a potential tool to explore COVID-19 propagation [84,85,86] and human small intestinal organoids serve to be an important model for the study of coronavirus infection as reported by *Lamers* et al. [87]. The authors showed that enterocytes were infected by SARS-CoV and SARS-CoV-2 and an increase in cytokine and interferon stimulated genes were associated with type I and III interferon responses [87]. Although organoids are useful to study bacterial monocultures [88,89] or culture with patient-derived microorganisms [90], maintaining these organoid cultures without bacterial contamination and/or major investments are the main challenges to this approach [91]. Since accessing organoids lumen is challenging, microinjection is used to deliver bacteria to organoids lumen effectively; however, this is technically difficult and a time-consuming process [92]. Therefore, in order to get access to different compartments of the organoids more effectively, 2D monolayers of organoids might be generated for these kinds of studies, e.g., grown on Transwell inserts [92]. For instance, intestinal stem cells released from enzymatically fragmented organoids from patient biopsies were plated into Transwell inserts to generate primary intestinal monolayers to accommodate sampling at both apical and basolateral side [93]. In addition, Roodsant et al. successfully studied microbial translocation (EV-A71 and *L. monocytogenes*) by using the 2D organoid model human fetal intestine [94]. This study validated the use of monolayer model as it preserves the epithelium characteristics; epithelial barrier, gene expression profiles, and epithelium polarization. However, model like this still lack characteristics like peristaltic flow as such to mimic the intestinal environment in vivo [95].

Despite the clear advantages of applying multi cell-type phenotypic intestinal organoids in comparison to single-cell lines, organoids still lack specific parts of the intestinal physiology, such as stroma, vasculature, immune system, and microbiome which limits the utility of organoids to completely recapitulate the in vivo situation [69]. Co-culturing organoids with other cell types is not well established and maintaining organoid cultures is more expensive than cell lines [96].

### 2.2. Ex Vivo Models

#### 2.2.1. Ussing Chamber

The Ussing chamber was first developed in the 1950s by Danish biologist Hans Ussing to understand the active Na^+^ transport in ex vivo intestinal tissue [97]. The chamber consists of two compartments separated by tissue or cell monolayers, thus isolating both apical and basolateral epithelium sides [54]. Electrodes measure voltages and short circuit current, and thereby, the Ussing chamber can measure permeability and transport of ions across epithelial tissues and cells such as the cystic fibrosis airway epithelia or human sinonasal epithelia [54,98,99]. A representative figure of this model is shown in Figure 1c. The use of this system paved the way towards the development of present models of trans-epithelial transport, which includes the discovery of Na^+^/K^+^ ATPase pump [100,101].

One such example of utilizing this approach for transport studies was the measurement of neurotransmitters, 5-hydroxytryptamine (5-HT) flux, and 5-HT uptake in mouse intestinal mucosa lacking a seromuscular layer [39]. In clinically relevant situations, such as cystic fibrosis, Ussing chamber has been used to measure ion transport function in rectal biopsies [102]. Furthermore, the Ussing chamber has been widely used to study gut integrity and intestinal permeability in both mouse intestinal inflammation models as well as human colonic biopsies [103]. Additionally, gut integrity can be measured in different regions of the colon in various abnormalities [103]. For instance, increased permeability in mid-colonic region of mice was observed and in human, the left colon showed more permeability as compared to the right colon with the help of Ussing chamber [103].

With respect to host–microbe interactions, studies on bacterial endotoxin response [104] and the mechanism of action of probiotics have been performed in Ussing chambers [105]. The effects of *Shigella* enterotoxin were shown first in rabbit intestine using Ussing chamber [106]. *Shigella* induced an increase in fluid and electrolyte accumulation and sodium secretion in rabbit ileal mucosa [106]. In addition, a commercially available probiotic, Bifico, improved epithelial barrier function in the colon of IL-10 depleted mice and reduced pro-inflammatory cytokine secretion [107]. In another study, Isenmann et al. demonstrated the effects of *Enterococcus faecalis* (*E.faecalis*) on bacterial invasion in the colonic mucosa of rats using the Ussing chamber [108]. The study showed bacterial translocation into colonic mucosa due to the aggregation product produced by *E.faecalis* [108].

Despite the many advantages of the system in measuring intestinal permeability, integrity, and transport in intact intestinal tissue, the use of Ussing chamber has several limitations. For instance, ex vivo tissue explants used in Ussing chambers are viable for a maximum period of 5 h and hence cannot be used for long term studies [54]. Low throughput and handling complexity is another major disadvantage of the system [109].

#### 2.2.2. Everted Sac

The everted gut sac model was developed in the 1950s by Wilson and Wiseman [110] to study the kinetics and mechanisms of drug absorption [111,112]. This model is mostly used to study drug metabolism, absorption, and the assessment of other pharmacokinetic parameters, such as multidrug resistance and drug interactions in the gastrointestinal segments [113]. The everted gut sac is prepared by segmenting part of the intestine (duodenum, jejunum, ileum, or colon) and everting the pieces of intestine washed by a physiological solution over a glass rod [113]. The sac is then filled with Krebs solution, sealed, and transferred to the incubation flask containing oxygenated media at 37 °C [113]. A representative figure of the model is illustrated in Figure 1d.

Everted gut sac experiments use intestinal tissue from different animals; rat is the most commonly used animal source [113,114]. This model has been used to determine the role of P-glycoprotein(P-gp) in transport of drugs used for diabetic patients like repaglinide (REG) in intestine [114], as well as to study P-gp-mediated efflux of anti-cancer agents using [^3^H]vinblastine, [^14^C]doxorubicin, and verapamil compounds [115]. This strategy has also proven to be useful in studying the intestinal permeability of artesunate-loaded solid lipid nanoparticles in rat jejunum [116]. The apparent permeability for nanoparticles was found to be greater compared to the pure artesunate [116]. As compared to cell lines this system shows higher paracellular transport of mannitol and it offers an inexpensive and relatively simple tool to study mechanisms related to drug absorption and kinetics across different regions of the intestine [112].

Although this model closely mimics gastrointestinal tract conditions, tissue viability is one of the main limiting factors [113]. The recommended tissue viability under physiological conditions is around 2 h [112]. Furthermore, this system is sensitive as it depends on various factors such as animal age, sex, species, intestinal region used, and other physiological factors such as pH and temperature [113]. Moreover, this model is not suitable for human intestine, limiting its translation to human physiology and disease. To the best of our knowledge, no microbial research is done till date using this system.

#### 2.2.3. InTESTine™ System

The Netherlands Organization for Applied Scientific Research (TNO) has recently developed an ex vivo tissue model called the InTESTine™ (Figure 1e). Like in the Ussing chamber, fresh intestinal tissue (duodenum, jejunum, ileum, colon) of human or porcine origin are mounted in the two-compartment system creating an apical and basolateral side [117]. As compared to Ussing chamber, which is widely used for permeability studies, the InTESTine™ system provides a higher throughput and easy horizontal setup in standard 6- or 24-well plates via which up to 96 ex vivo intestinal tissue can be used per day to test for intestinal absorption [109]. For instance, porcine intestinal tissue was used to determine the permeability of drugs such as atenolol, mannitol, cimetidine, and caffeine from duodenum to ileum where the permeability of atenolol remained stable while a significant increase in permeability was seen in mannitol, cimetidine, and caffeine from duodenum to ileum [109]. In addition, the tissue mounting is done horizontally so that the system can be incubated at 37 °C on a rocker, thereby reducing foaming and possible evaporation while simultaneously enabling direct contact of the epithelial tissue with the target compounds [109,117].

The system is designed to study the translocation and absorption of biological, nutritional, or pharmaceutical compounds across the intestinal wall in a physiologically relevant manner [109,118]. It is also suitable to study complex intestinal processes due to the presence of multiple cell types and layers in the ex vivo tissue [109,118]. For example, the satiety hormones endocrine responses were determined after stimulation with rebaudioside A and casein which resulted in increased luminal and basolateral side secretion of GLP-1 and PYY [118]. In addition, testosterone metabolism by CYP3A4 was shown and regarding host–microbe interactions, co-incubation with a probiotic strain, *Lactobacillus rhamnosus* increased tissue viability [118], which shows the system’s versatility to study diverse GI functions.

When using porcine intestinal tissue in the system, a good characterization of the tissue is needed in terms of the metabolic and transport capacity to have a good prediction for the human situation [56,109]. Furthermore, as for Ussing chamber, the major limitation of working with ex vivo tissue in this system is the limited tissue availability and viability [117]. In order to extend the tissue viability, these researchers have adapted the InTESTine model into a microfluidic model called the Intestinal Explant Barrier Chip (IEBC) which has so far been used for studies up to 24 h [119].

### 2.3. Microfluidic Gut-On-Chip Models

Microfluidic gut-on-chip models have emerged as a novel approach to study intestinal functions through incorporating multiple cell types and/or gut microbiome into the system [120,121,122]. In the conventional 2D and 3D systems tissue-tissue interfaces are difficult and the static nature of Transwell inserts or cell culture systems is opposing the living intestinal environment, which should allow a constant flow of nutrients and oxygen supply and removal of waste residues [93,123]. Using microfluidic flow to supply and remove medium, the microphysiological gut-on-chip systems aim to overcome these limitations and more closely reflect the intestinal microenvironment [124,125]. In general, most gut-on-chip models have two microchannels that are separated by a semi-permeable porous membrane made up of different array of materials of varying pore sizes and the membrane is seeded with gut epithelial cells [126,127,128,129]. The pores in the membrane support transport of soluble molecules between the channels [129].

**Figure 2:**
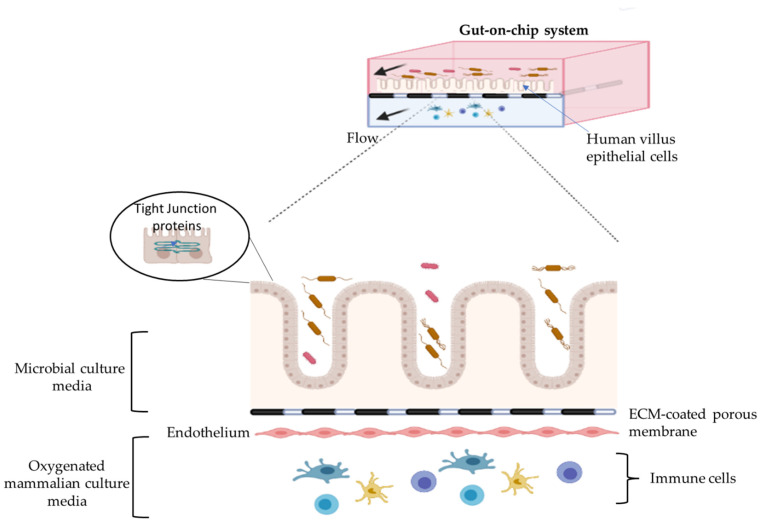
This illustration describes an example of gut-on-chip system. Epithelial cells on the chip differentiate to form villi, and polarize to form microvilli on the apical side. In this system, epithelial cells can be cultured in direct exposure with intestinal microorganisms, nutrients, drugs, or metabolites. In some systems, endothelial cells are grown on the basolateral side of the ECM-coated membrane. This system guarantees a continuous flow of oxygen on the basolateral side by using oxygenated mammalian-cell culture medium, whereas it is possible to use other types of (anaerobic) culture media on the apical side of the system. Immune cells, metabolites and other features can be introduced in the model to mimic, for instance, specific disease situations. This illustration was created with BioRender.com.

Typically, intestinal epithelial cells, like Caco-2 or patient-derived cells, are seeded into the device and allowed to adhere for a short while, followed by the media that is pumped through the channels via syringe or peristaltic pumps in order to create flow [123,129,130]. The flow can also be gravity driven as seen in a multi-well plate platform like the OrganoPlate^®^ (Mimetas BV, Leiden, The Netherlands) that therefore needs to be incubated on a rocker platform with tilting functionalities [131,132]. With flow, intestinal cells spontaneously differentiate and form a 3D villi-like structure [133]. The epithelial cells lining the villus structure are also covered by brush border and mucus, and are linked by tight junctions, thus recapitulating the structure and function of the normal human intestinal villi [133]. Through vacuum application to hollow side chambers of one of the available gut-on-chip models, peristaltic motions can be created [123,134]. In addition, some advanced chip devices offer the possibility to seed endothelial cells on the basolateral side of the membrane [135] and/or include immune cells in the basolateral chamber [136]. Some characteristics of the gut-on-chip system are summarized in Figure 2. As well as cell lines, organoids are also applied in microfluidic chip systems [137,138]. In addition, some research groups developed tissue-based platforms in which intact intestinal tissue can be studied on gut-on-chip devices [119,139,140,141,142]. For instance, rat intestinal tissue to which microfluidics was applied showed metabolic activity and retention of tissue viability up to 8 h [139]. Additionally, intestinal tissue from patients undergoing small or large bowel resections were used to study tissue viability and integrity for up to 72 h validating the use of tissue in a microfluidic set-up to investigate the pathophysiological condition and/or new drug interactions in the diseased situation [140].

Generally, the cells or tissue use in gut-on-chip devices can be probed from both apical and basolateral sides to measure barrier function [143,144] and drug and nutrient absorption [145,146]. In most cases the gut-on-chip device is made of polydimethylsiloxane (PDMS) as it is user-friendly, cheap, has a non-toxic surface, and is permeable to gasses [147,148]. To name a few of these PDMS-based gut-on-chips: (Small) Intestine Chip consisting of duodenum-derived organoids [135,137], Jejunum-Intestine-Chip with jejunal human enteroids [149], Gut Chip [123], and Colon Chip [150]. There are also examples of 3D printed chips [151] or chips made of other materials like glass [152] or lipophilic coatings [153]. In a more sophisticated gut-on-chip model, human intestinal epithelium, immune cells, capillary endothelium, and commensal microbes functionally co-exist and interact with one compartment representing the gut lumen whereas the other represents blood vessels lined with vascular endothelium [120,129]. An example of such a specific gut-on-chip model was used to study the effects of radiation therapy on intestinal epithelium [135]. γ-radiation treatment increased reactive O_2_ species, DNA fragmentation, cytotoxicity, apoptosis, and compromised intestinal barrier integrity [135]. In this model dimethyloxaloylglycine was identified as prophylaxis in suppressing adverse effects of radiation therapy [135]. Assessment of the epithelial barrier in the gut-on-chip models is frequently done by measuring the permeability of FITC-labeled dextran of high molecular weight [140,142]. Another possibility to assess barrier function is to measure TEER by placing electrodes on opposite sides of the established barrier [154,155]. Once the intact barrier is established, intestinal functions can be studied. For instance, a gut-on-chip platform was used with a microhole-trapping array instead of the membrane structure to determine cellular permeability of diuretics using Caco-2 cells [156]. The permeability of 10 drugs was measured in this model and to found to be correlated with the in vivo observations in humans [156].

Some gut-on-chip devices include microbes in the apical channel to study host–microbe (immune) responses. As an advantage over static in vitro cell-based models in which bacteria and other microbes are known to rapidly overgrow intestinal cells, microfluidic flow in the gut-on-chip devices allows the formation of a protective mucus layer, and oxygen gradient thereby supporting the co-culture of cells and microbes for longer periods [133,137,157,158,159]. For example, a gut-on-chip co-culture of Caco-2 cells with the commensal intestinal microbe, *Lactobacillus rhamnosus GG*, showed an improved barrier function similar to observations in vivo in humans [123]. In addition, this model is used to study Coxsackie B1 virus pathogenesis, an enteric virus infection in vitro [160]. Another gut-on-chip device that was lined with human colonic epithelial cells derived from patient-derived organoids highlighted how human microbiome derived metabolites were responsible for higher susceptibility to enterohemorrhagic *Escherichia coli* (EHEC) infection [150]. Although anaerobic bacteria account for the biggest part of our gut microbiome, only a few studies included these anaerobes with cells for a longer period of time. One example includes the use of an anaerobic gut-on-chip model to study direct interaction between bacteria and Caco-2 cells, where, in particular, obligate anaerobes were cultured with Caco-2 cells for up to 5 days [161].

With respect to intestinal disease, the gut-on-chip model has been used to study intestinal inflammation, especially in the context of IBD. One study showed decreased barrier integrity and increased inflammation in Caco-2 and HT-29-MTX-E12 tubuli upon pro-inflammatory cytokine (TNF-α and IL-1β) treatment in the apical and basolateral channel of the OrganoPlate^®^ (Mimetas BV, Leiden, The Netherlands) containing 40 microfluidic chips, in combination with THP-1 and MUTZ-3 immune cells [136]. Furthermore, a two-day treatment of Caco-2 cells in another microfluidic chip with 2% DSS resulted in a decreased barrier integrity as reflected by TEER and FITC-dextran permeability measurements, as wells as disrupted mucus barrier and reduced villus height [162]. A simultaneous pre-treatment with eight probiotic bacterial strains helped to maintain the intestinal barrier but not when the epithelium was exposed to DSS before the treatment with the probiotics [162].

Despite the interesting possibilities with this model, there are several limitations. Cell- and organoid-based gut-on-chip models lack different cell types which remains still a hurdle with respect to an adequate representation of the intestinal architecture and microenvironment [120]. Further developments of 3D bio-printing technologies may prove to be useful in the precise reproduction of complex and interconnected structure as seen in vivo [163,164,165]. Furthermore, the platform is complex and labor intensive and the throughput is low. Reproducibility and robustness of the model in different laboratories is another concern [120]. Additionally, when it comes to materials used in such systems, they should offer biocompatibility, non-leaching, inert and non-adsorbent properties. For example, the frequently used PDMS can adsorb small molecules, drugs or other fluorescent markers that are present in the medium and thereby reduce the reproducibility of solute molecules concentration [166,167].

## 3. Conclusions

Over the last decades, various in vitro and ex vivo models have been developed and utilized to study the gastrointestinal tract’s physiology, pathology, and pharmacology. Yet a complete understanding of human intestinal physiology and function is still largely unknown. The various intestinal research models available are focused on the intestinal environment. However, each system has its advantages and disadvantages (Table 1), be it high throughput, cost-effectiveness, co-culture, or tissue availability therefore demanding a careful evaluation to which system or technique best fit the research purposes. In this review, we summarized different approaches to study the various intestinal functions, these in vitro and ex vivo approaches might reduce the need for animals in research. Approaches such as Ussing chamber, everted sac, organoids, and the different cell lines in use are promising; however, they do not completely mimic the physiologically relevant in vivo situation in human mainly due to the complex intestinal environment or challenges involving tissue viability, risk of contamination etc. The recently developed, state of the art gut-on-chip model has progressed in answering some of the questions about mimicking the natural intestinal physiology, however the improvements on the existing system with the advent of 3D bio-printing and use of alternative matrix materials might be useful in guiding future research in this field. Moreover, an advanced understanding of these various approaches will yield a better experimental design for different questions in intestinal research.

## Figures and Tables

**Table 1:** Advantages and disadvantages of intestinal in vitro and ex vivo models.

Models	Advantages	Disadvantages
Cell lines (Caco-2, HT-29, T84) [13,54,55,56,57]	Commercially available Can be polarized Cost effective High throughput Robust	Cancerous origin Difficult to control differentiation in Caco-2 cells HT-29 shows impaired glucose metabolism Monocellular phenotype Lacks certain cells/tissue formation
Organoids [65,74,75,91,96]	Cell type diversity Used in both 2D-3D forms Can be polarized as monolayer Robust Patient-derived organoids can be used for precision medicine such as IBD and cystic fibrosis	High costs Access to lumen remains challenging Labor-intensive Lacks certain cells/tissue formation Random and uncontrolled growth even from the same stem cell lines
Ussing chamber [54,103,109]	Uses intact tissue Multiple intestinal regions Set-up with electrodes to measure TEER and ion transport Two compartments	Labor-intensive Limited tissue viability (maximum 5 h) Limited availability of human tissue Low throughput
Everted Sac [112,113,114]	Intact intestinal tissue Large surface area for absorption Presence of mucus layer	Mostly used with rat tissue and therefore less relevant for humans Labor-intensive Limited tissue viability (2 h)
InTESTine™ [109,117]	Intact intestinal tissue Multiple intestinal regions Two compartments Horizontal orientation of the tissue Fits in standard 6- or 24-well plates Higher throughput as compared to Ussing chamber Easy to handle Up to 96 intestinal tissues can be used per system each day	Limited tissue availability and viability
Microfluidic gut-on-chip [120,123,124,129,133]	Mechanical stress on the cells/tissue Continuous supply of fresh nutrients and removal of waste products Faster differentiation of cells Appearance of villi-like structures Supports the formation of mucus layer and allows co-culture of cells and microbes	High costs Complex Labor-intensive Low throughput
